# Dialing into health: assessing the reach, use, and impact of mobile phones on health awareness among rural women in the Rural Health Training Centre area of District Gautam Buddha Nagar, Uttar Pradesh, India

**DOI:** 10.1186/s12913-026-15008-w

**Published:** 2026-06-20

**Authors:** Varun Pal, Ishant Kumar, Kabita Barua, Meraj Gohar, Shalini Srivastava, Harsh Mahajan, Neeraj Pal Singh, Rahul Chaudhary, Tashika Bansal, Tanisha Gupta, Tauqir Ahmed, Tanya Singhal

**Affiliations:** 1 School of Medical Sciences and Research, Sharda University, Knowledge Park III, Greater Noida, Uttar Pradesh 201310 India; 2 Department of Community Medicine, School of Medical Sciences and Research, Sharda University, Knowledge Park III, Greater Noida, Uttar Pradesh 201310 India

**Keywords:** Mobile health, Rural women, Digital engagement, Health communication, India

## Abstract

**Background:**

Mobile health (mHealth) interventions are increasingly promoted to strengthen health communication in rural India. However, evidence remains limited on how rural women translate mobile phone access into meaningful health-related use and trusted engagement, creating challenges for designing equitable digital health strategies.

**Methods:**

A community-based cross-sectional study was conducted among 374 rural women aged ≥ 18 years in five villages under the Rural Health Training Centre area of Gautam Budh Nagar, Uttar Pradesh. Multistage random sampling was employed. Data were collected using a pre-tested structured questionnaire capturing sociodemographic characteristics, mobile phone ownership and access, health-related usage, trust in mobile-delivered information, preferences for information delivery, and perceived barriers. Descriptive statistics summarized patterns, chi-square tests assessed associations, and multivariable logistic regression identified independent predictors of health-related mobile phone use.

**Results:**

Among participants, 51.1% were aged 18–31 years, and 79.7% belonged to the middle socioeconomic class. Smartphone ownership was reported by 54.5% (95% CI: 49.5–59.6), while 35.8% (95% CI: 31.0-40.7) relied on borrowed devices and 2.7% (95% CI: 1.3–4.8) had no mobile access. Despite this, 52.4% (95% CI: 47.2–57.5) reported never using mobile phones for health purposes, and only 3.2% (95% CI: 1.7–5.5) reported consistent use. Trust in mobile health information was moderate, with 66.8% (95% CI: 61.8–71.6) reporting occasional trust. After adjustment, education remained a significant predictor of health-related mobile phone use; women with primary or middle education (AOR = 0.28; 95% CI: 0.14–0.55) and those with no formal education (AOR = 0.20; 95% CI: 0.10–0.39) were less likely to report health-related use compared to graduates. Voice calls were the most preferred communication mode, and lack of knowledge was the most frequently reported barrier.

**Conclusion:**

Although mobile phone access among rural women is relatively widespread, meaningful health-related engagement remains limited. Interventions focusing on digital literacy, voice-based communication strategies, and sociocultural barriers are essential to enhance the effectiveness of mHealth programs in rural settings.

**Supplementary Information:**

The online version contains supplementary material available at https://doi.org/10.1186/s12913-026-15008-w.

## Introduction

The rapid expansion of mobile technologies has transformed health communication globally, offering new opportunities to improve health awareness, service utilization, and self-management of health conditions. Mobile health (mHealth) interventions-including voice calls, short message services, and mobile applications-have shown potential to overcome traditional barriers to healthcare access, particularly in low- and middle-income countries (LMICs) where health systems face constraints related to workforce shortages, geographic inaccessibility, and limited infrastructure [1–3]. Evidence from systematic reviews and meta-analyses indicates that mHealth interventions can positively influence health behaviors and service uptake, especially in maternal, newborn, and child health domains [4–7].

India, with one of the world’s largest mobile subscriber bases, has actively promoted mHealth as a health-system strengthening strategy. National initiatives such as Kilkari, which delivers stage-specific maternal and child health information through voice calls, and Mobile Academy, which provides digital training for frontline health workers, illustrate policy-level commitment to digital health [19, 20]. However, the benefits of mHealth are not equitably distributed. Nationally representative data from the National Family Health Survey (NFHS-5) show that only about half of Indian women aged 15–49 years personally own and use a mobile phone, with substantially lower access among rural women compared to their urban counterparts [21]. An NFHS-based analysis further estimates that women’s personal access to mobile phones in rural India remains around 40–45%, underscoring a persistent gendered digital divide despite high overall household phone penetration [17].

Existing Indian studies have documented associations between mobile phone access and improved awareness or utilization of specific health services, particularly in maternal and child health. However, much of this literature focuses either on technological access or on the effectiveness of specific mHealth interventions. There remains comparatively limited evidence that examines, in an integrated manner, how rural women navigate mobile phone ownership versus borrowed access, how frequently phones are used for health purposes, how much trust women place in mobile-delivered health information, and which modes of communication they find most acceptable. While several studies have explored mobile phone ownership or evaluated specific mHealth interventions in India, these investigations often assess individual components of digital health engagement in isolation rather than examining how access, usage behavior, trust, and perceived benefit interact within real-world rural contexts [12, 16, 18]. This fragmented understanding presents a significant challenge for program planners and policymakers, because digital health strategies are frequently designed based on assumptions of device availability without sufficient attention to patterns of shared access, privacy constraints, digital literacy limitations, and sociocultural norms affecting women’s independent use of technology [14, 15].

The absence of integrated evidence is particularly problematic because mobile health programs that rely solely on indicators such as household phone penetration may overestimate effective reach and engagement among rural women. Without understanding how women actually use and trust mobile technologies, interventions risk being underutilized, misaligned with user preferences, or inequitably distributed across population groups. Furthermore, inadequate insight into preferred communication modes and perceived barriers may lead to inefficient allocation of resources and limited scalability of mHealth interventions. Addressing this knowledge gap is therefore essential for designing context-sensitive digital health strategies that are both accessible and acceptable to rural women, particularly in regions where gendered digital divides persist [17, 21].

Trust and acceptability are particularly important in rural contexts, where interpersonal communication through frontline health workers such as Accredited Social Health Activists (ASHAs) and Auxiliary Nurse Midwives (ANMs) continues to be the most credible source of health information [10, 12]. While programmes like Kilkari are designed to reach women directly through voice calls-thereby addressing literacy barriers-their actual reach and impact depend on accurate registration, stable phone numbers, and consistent access to devices, which may be compromised when women rely on borrowed phones or have limited control over device use [20]. Without understanding these contextual realities, digital health initiatives risk suboptimal uptake, inefficient use of resources, and the potential widening of existing health and digital inequities.

Against this background, the present study was undertaken to assess the reach, use, and perceived impact of mobile phones on health awareness among rural women in the Rural Health Training Centre (RHTC) area of Gautam Buddha Nagar district, Uttar Pradesh. Specifically, the study examined three key dimensions: (1) reach, defined as patterns of mobile phone ownership and access; (2) use, defined as health-related utilization of mobile phones and levels of digital engagement; and (3) impact, defined as perceived improvements in healthcare access, preferences for receiving health information, and barriers influencing mobile phone use. By structuring the investigation across these interconnected domains, the study aimed to generate implementation-relevant insights for designing equitable and context-appropriate mHealth strategies for rural women in India.

## Methodology

### Study design and setting

A community-based cross-sectional study was conducted among rural women residing in the field practice area of the Rural Health Training Centre (RHTC), Gautam Budh Nagar district, Uttar Pradesh. Data collection was undertaken over 6 months, from June 2025 to November 2025.

Uttar Pradesh provides a particularly relevant setting to examine these issues. As India’s most populous state with a large rural population, Uttar Pradesh continues to face challenges related to women’s education, digital access, and health service utilization. NFHS-5 state-level data indicate that women’s personal mobile phone use in Uttar Pradesh remains below national urban averages, making it a critical context for assessing real-world engagement with mobile-based health information [22]. Studying rural women in an area served by a Rural Health Training Centre (RHTC) allows exploration of mHealth engagement in a setting with functional primary healthcare services, while still reflecting common rural constraints faced by women in similar contexts.

The RHTC area was selected because it represents a typical rural population of Western Uttar Pradesh in terms of sociodemographic characteristics, health service availability, and digital access patterns. Although communities attached to training centres may have greater exposure to routine health activities, service delivery in this area is largely comparable to other rural primary healthcare settings. Therefore, findings are likely to be generalizable to similar rural contexts with functional primary health infrastructure, while caution is warranted when extrapolating to remote or underserved areas lacking such facilities.

### Study population

The study population comprised rural women aged 18 years and above who had been residing in the selected villages for at least six months and irrespective of mobile phone access status, either through personal ownership or by borrowing a family member’s device. Women who were below 18 years of age or declined to provide consent were excluded from the study. Including all women, regardless of mobile phone access, allowed identification of barriers related to non-use and enabled estimation of the true reach and engagement of mobile-based health communication in the study population.

### Sample size

The sample size was calculated using the standard formula for prevalence studies [23]:

$$n\, = \,\left({{Z^2}\, \times \,p\, \times \,q} \right){\rm{ }}\,/\,{d^2}$$

where Z = 1.96 (95% confidence level), *p* = 41.6% (estimated prevalence of women’s personal access to a mobile phone based on previous literature) [17], q = 1 − p, and d = 5% precision. The minimum required sample size was calculated as 374 participants.

### Sampling procedure

A multistage random sampling technique was employed. In the first stage, five villages were randomly selected from the total villages covered under the RHTC area. In the second stage, a complete household list for each selected village was obtained from ASHA registers and village records. Based on village records and ASHA registers, the estimated number of eligible women in these villages was approximately 2384. Systematic random sampling was then used to select households from each village proportional to village size. Within each selected household, all resident women were screened for eligibility. If more than one eligible woman was present in a household, one participant was selected using a simple random (lottery) method to avoid selection bias. Only one woman was interviewed per household. A total of 374 eligible women were selected using systematic random sampling, and all selected participants consented to participate in the study, resulting in a response rate of 100%.

### Conceptual basis of the study

The study was guided by a conceptual model in which mobile phone access (personal ownership or borrowing) influences health-related use, which in turn shapes trust in mobile-delivered health information and perceived impact on healthcare access [3]. Sociodemographic characteristics were conceptualized as upstream determinants influencing each of these domains. Although no formal implementation framework (e.g., RE-AIM) was applied, the questionnaire was structured around key constructs of reach (access), engagement (use), acceptability (trust), and perceived benefit (impact). These constructs were explicitly aligned with the study objectives to ensure that data collection, statistical analyses, and interpretation of findings remained focused on evaluating the reach, use, and impact of mobile phone-based health communication.

### Study tool and data collection

Data were collected using a structured, pre-tested questionnaire administered through face-to-face interviews by trained field investigators after obtaining verbal informed consent. Privacy and confidentiality were ensured during interviews. The questionnaire was developed based on a review of relevant literature and consultation with public health experts. It was pre-tested among 20 rural women from a non-study village to assess clarity, cultural appropriateness, and flow. Minor modifications were made based on feedback, and data from pretesting were not included in the final analysis. The questionnaire included sections on Sociodemographic characteristics, Mobile phone ownership and access, Use of mobile phones for health-related purposes, Trust in mobile-delivered health information, Preferred modes of receiving health information, Perceived barriers to using mobile phones for health communication, Perceived impact of mobile phone use on access to healthcare.

### Operational definitions

Mobile phone ownership was defined as personal possession of a mobile phone (smartphone or feature phone). Mobile phone access referred to the ability to use a mobile phone either through personal ownership or by borrowing a family member’s device.

Health-related mobile phone use referred to the use of a mobile phone to seek, receive, or manage information related to health needs such as maternal health, child care, or chronic conditions.

Socioeconomic status was assessed using the BG Prasad socioeconomic scale (2025), based on per-capita monthly income calculated by dividing total family income by the number of family members and categorizing households into upper, middle, and lower classes [24]. 

Health-related mobile phone use was assessed among all enrolled participants, irrespective of whether they personally owned a mobile phone, borrowed one, or had no access, in order to capture the full spectrum of health communication behavior.

### Data analysis

Data were entered into Microsoft^®^ Excel Version 16.93.1 (25011917) and analyzed using Statistical Package for Social Sciences, Version 31.0.0.0 (117), IBM, Chicago, USA. Descriptive statistics, including frequencies, percentages, means, and standard deviations, were used to summarize participant characteristics and key study variables. Bivariate associations between sociodemographic variables and outcomes were examined using the Chi-square test.

Bivariate associations between sociodemographic characteristics and study outcomes were examined using the Chi-square test. Binary logistic regression analysis was performed to identify independent predictors of health-related mobile phone use, which represented the principal behavioral outcome under the “use” domain of the study. For regression analysis, health-related mobile phone use was recategorized into a binary outcome variable, where participants reporting “always” or “sometimes” using mobile phones for health purposes were classified as “Yes,” and those reporting “never” were classified as “No.”

Variables included in multivariable regression models were selected based on theoretical relevance, prior evidence from published literature, and potential confounding value, rather than solely statistical significance in bivariate analyses, consistent with epidemiological modeling principles [4]. Age, education, socioeconomic status, marital status, and mobile phone ownership were included in the adjusted model. Family type and occupation were initially explored in bivariate analyses but were not retained in the final model because their inclusion did not materially improve model performance or alter effect estimates.

Results are presented as adjusted odds ratios (AORs) with 95% confidence intervals.

A composite digital engagement score was developed by the authors to summarize overall engagement with mobile phone-based health communication. The score incorporated three domains: mobile phone access (0–3 based on level of access), health-related mobile phone use (0–2 based on frequency of use), and trust in mobile-delivered health information (0–2 based on degree of trust), yielding a total score ranging from 0 to 7. Higher scores reflected greater digital engagement. The score was used as a descriptive and comparative index rather than a validated standardized scale.

Figures were generated using SPSS, Python’s Matplotlib library (version 3.x) and Microsoft Excel and exported in high-resolution formats suitable for publication.

### Ethics approval and consent to participate

The informed consent was obtained from all the participants. This study was conducted following the Declaration of Helsinki and was approved by the Institute’s Ethical Committee (Ref. No. SU/SMS&R/76-A/2025/234) of School of Medical Sciences and Research, Sharda University, Knowledge Park III, Gautam Buddha Nagar, Greater Noida, 201,310, India. No identifiable personal information was collected to ensure confidentiality. The participants were informed of their right to withdraw from the study at any point they deemed necessary, without any consequences and the information was used solely for academic and improvement purposes.

## Results

Of the 374 eligible women approached, all consented to participate, resulting in a response rate of 100%. A total of 374 rural women were included in the analysis. The results are presented according to domains corresponding to the study objectives of reach, use, and impact, thereby ensuring alignment between the research question, analytical approach, and presentation of findings.

### Sociodemographic profile of participants

The sociodemographic characteristics of the study participants are summarized in Table 1. Over half of the respondents were aged 18–31 years, and the majority were married. Most participants belonged to Hindu households and lived in joint families. More than half had education up to the primary or intermediate level, while nearly one-third had no formal education. Most women were not engaged in paid employment. Based on the BG Prasad socioeconomic classification (2025), most participants belonged to the middle socioeconomic category, with smaller proportions in the upper and lower classes.

**Table 1:** Sociodemographic characteristics of the study participants (*N* = 374)

Variable	Category	Frequency (*n*)	Percentage (%)
Age group (years)	18–31	191	51.1
	32–45	118	31.6
	≥ 46	65	17.4
Marital status	Married	329	88.0
	Unmarried	45	12.0
Religion	Hindu	361	96.5
	Muslim	13	3.5
Family type	Joint	218	58.3
	Nuclear	111	29.7
	Extended	45	12.0
Education level	Graduate/Postgraduate	66	17.6
	Secondary (High school/Intermediate)	122	32.6
	Primary/Middle school	74	19.8
	No formal education	112	29.9
Occupation	Employed	31	8.3
	Homemaker/Unemployed	343	91.7
Socioeconomic status *(BG Prasad 2025)*	Upper	69	18.4
	Middle	298	79.7
	Lower	7	1.9

### Mobile phone access and ownership (reach)

Patterns of mobile phone access and ownership among study participants are summarized in Table 2. More than half of the women reported personal smartphone ownership **(**54.5%; 95% CI: 49.5–59.6), while over one-third relied on borrowing a family member’s mobile phone **(**35.8%; 95% CI: 31.0-40.7). A smaller proportion reported owning feature phones **(**7.0%; 95% CI: 4.6–9.9), and only a minimal proportion reported having no access to a mobile phone (2.7%; 95% CI: 1.3–4.8). One response for health-related mobile phone use were missing due to incomplete responses and were retained as missing values during analysis. More than half of women reported personal smartphone ownership, while over one-third relied on borrowed phones. More than half of women reported personal smartphone ownership, while over one-third relied on borrowed phones.

**Table 2:** Mobile phone access, health-related use, and trust indicators among rural women (*N* = 374)

Variable	Category	*n*	%	95% Confidence Interval
Mobile phone access	Smartphone ownership	204	54.5	49.5–59.6
	Borrowed phone access	134	35.8	31.0-40.7
	Feature phone ownership	26	7.0	4.6–9.9
	No mobile access	10	2.7	1.3–4.8
Health-related mobile phone use	Always	12	3.2	1.7–5.5
	Sometimes	164	43.9	38.8–49.1
	Never	197	52.7	47.5–57.9
	Missing response	1	0.3	-
Trust in mobile health information	Always	44	11.8	8.6–15.5
	Sometimes	250	66.8	61.8–71.6
	Never	80	21.4	17.3–25.8

### Health-related mobile phone use and digital engagement (use)

Health-related use of mobile phones and digital engagement patterns across sociodemographic characteristics are presented in Table 3. Health-related use of mobile phones and digital engagement patterns across sociodemographic characteristics are presented in Table 3. Variation in health-related mobile phone use and digital engagement scores was observed across education and socioeconomic groups.

**Table 3:** Bivariate analysis of sociodemographic characteristics with health-related mobile phone use and digital engagement score (*N* = 374)

Characteristics	*n*	Health-related mobile phone use	*p*-value	Digital engagement score	*p*-value
		Always *n* (%)	Sometimes *n* (%)	Never *n* (%)	
Age group (years)					
18–31	191	8 (4.2)	92 (48.2)	91 (47.6)	0.041
32–45	118	3 (2.5)	48 (40.7)	67 (56.8)	
≥ 46	65	1 (1.5)	24 (36.9)	40 (61.5)	
Marital status					
Married	329	11 (3.3)	141 (42.9)	177 (53.8)	0.518
Unmarried	45	1 (2.2)	23 (51.1)	21 (46.7)	
Religion					
Hindu	361	12 (3.3)	158 (43.8)	191 (52.9)	0.734
Muslim	13	0 (0.0)	6 (46.2)	7 (53.8)	
Family type					
Joint	218	7 (3.2)	95 (43.6)	116 (53.2)	0.612
Nuclear	111	4 (3.6)	48 (43.2)	59 (53.2)	
Extended	45	1 (2.2)	21 (46.7)	23 (51.1)	
Education					
Graduate/Postgraduate	66	6 (9.1)	38 (57.6)	22 (33.3)	< 0.001
Secondary	196	5 (2.6)	85 (43.4)	106 (54.1)	
Illiterate	112	1 (0.9)	41 (36.6)	70 (62.5)	
Occupation					
Employed	31	2 (6.5)	15 (48.4)	14 (45.1)	0.091
Unemployed	343	10 (2.9)	149 (43.4)	184 (53.6)	
Socioeconomic status (BG Prasad)					
Upper	69	5 (7.2)	36 (52.2)	28 (40.6)	0.003
Middle	298	7 (2.3)	126 (42.3)	165 (55.4)	
Lower	7	0 (0.0)	2 (28.6)	5 (71.4)	

### Digital engagement score distribution

**Fig. 1:**
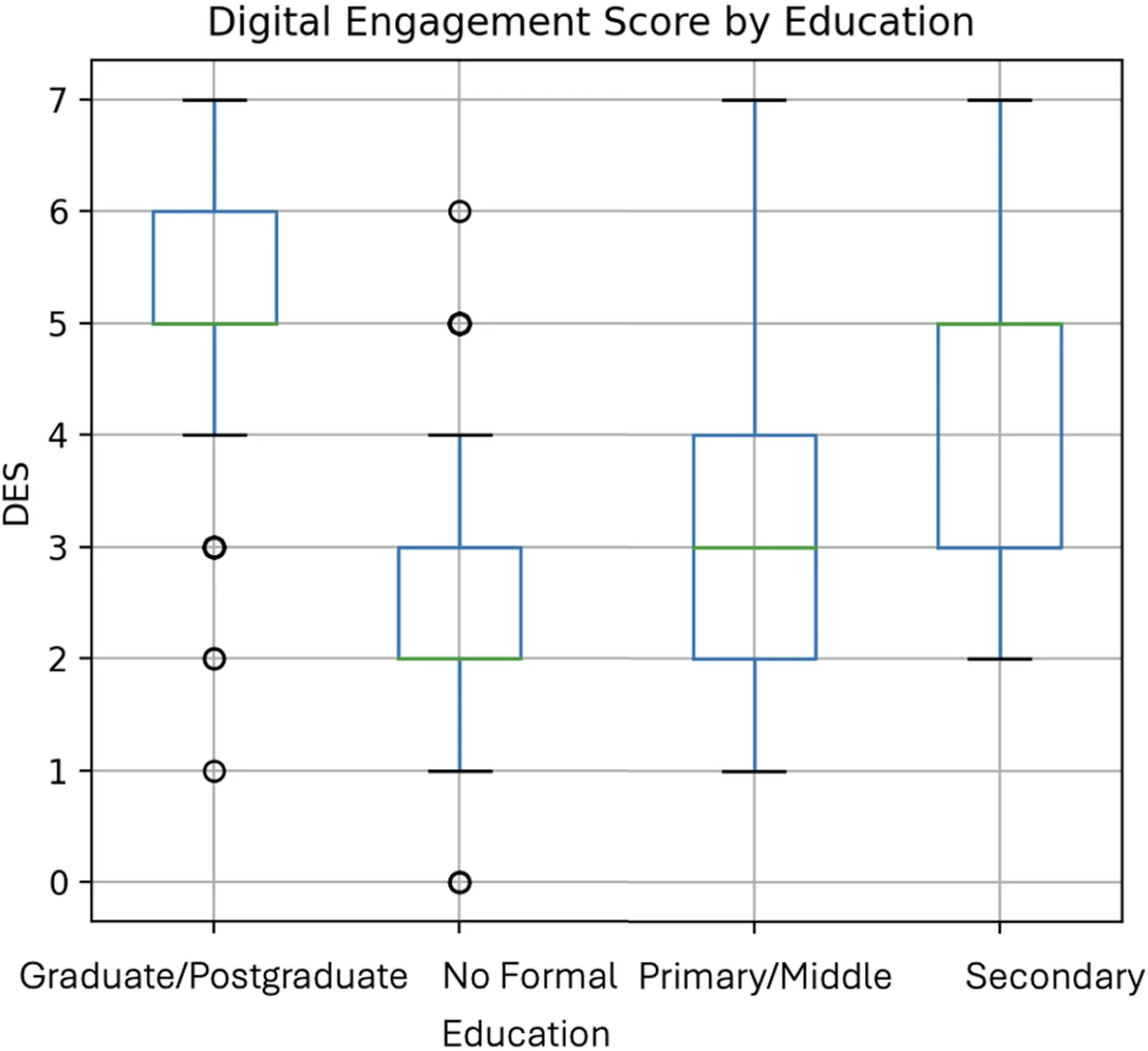
Distribution of digital engagement score across education levels

To capture overall engagement beyond individual indicators, a composite digital engagement score was constructed incorporating mobile phone access, health-related use, and trust in mobile-delivered health information. Figure 1 illustrates the distribution of digital engagement scores across education groups.

### Predictors of health-related mobile phone use

Multivariable logistic regression analysis was performed to identify factors associated with health-related mobile phone use among rural women (Table 4). After adjusting for age, marital status, socioeconomic status, and mobile phone ownership, education level remained a significant predictor of health-related mobile phone use. Compared to women with graduate or postgraduate education, those with secondary education had significantly lower odds of using mobile phones for health purposes (AOR = 0.41; 95% CI: 0.21–0.78; *p* = 0.007), while women with primary or middle-level education (AOR = 0.28; 95% CI: 0.14–0.55; *p* < 0.001) and those with no formal education (AOR = 0.20; 95% CI: 0.10–0.39; *p* < 0.001) had substantially lower likelihood of health-related mobile phone use. In contrast, age group, marital status, and socioeconomic status were not found to be statistically significant predictors after adjustment, although women with personal mobile phone ownership demonstrated higher odds of health-related use compared to those without personal ownership.

**Table 4:** Multivariable logistic regression analysis showing factors associated with health-related mobile phone use among rural women

Variable	Category	Adjusted Odds Ratio (AOR)	95% CI	*p*-value
Age group (years)	18–31	Reference	-	-
	32–45	0.88	0.54–1.42	0.60
	≥ 46	0.71	0.39–1.28	0.25
Education level	Graduate/Postgraduate	Reference	-	-
	Secondary	0.41	0.21–0.78	0.007
	Primary/Middle	0.28	0.14–0.55	< 0.001
	No formal education	0.20	0.10–0.39	< 0.001
Socioeconomic status	Upper	Reference	-	-
	Middle	1.12	0.68–1.86	0.64
	Lower	0.89	0.42–1.91	0.77
Marital status	Unmarried	Reference	-	-
	Married	1.26	0.72–2.18	0.42
Mobile phone ownership	No personal phone	Reference	-	-
	Personal ownership	2.34	1.48–3.69	< 0.001

### Preferences for information delivery and perceived impact

Preferences for receiving health-related information through mobile communication are summarized in Supplementary Figure S1. Voice calls were identified as the most preferred mode of communication, reported by 42.8% of participants, followed by door-to-door communication and text-based messaging. A smaller proportion of respondents expressed preference for video or audio-based communication formats. These findings suggest that although for interactive and voice-based communication channels over text-based approaches, particularly in populations with varying literacy levels.

Perceived impact of mobile phone use on healthcare access was assessed among women who reported any health-related mobile phone use. Among these participants, 109 out of 176 women (61.9%; 95% CI: 54.4–69.0) reported that mobile phone use had improved their access to healthcare services, while 67 (38.1%; 95% CI: 31.0–45.6) reported no perceived improvement. These findings suggest that although overall health-related use remained limited, a substantial proportion of users perceived measurable benefits in terms of improved communication, timely information, and easier access to health-related services.

### Barriers to using mobile phones for health communication

The most commonly reported barriers to mobile phone use for health communication included lack of knowledge (27.5%), language difficulties (6.8%), and family restrictions (4.4%). Technical constraints such as lack of network coverage (1.4%) and cost of data (0.8%) were reported less frequently. These findings are presented in detail in Supplementary Table S1.

## Discussion

This study examined the reach, use, and perceived impact of mobile phone–based health communication among rural women in Western Uttar Pradesh. The findings demonstrate that although mobile phone access was common, regular use of mobile phones for health-related purposes remained limited. Educational attainment emerged as the most consistent determinant of health-related mobile phone use, while preferences for voice-based communication and reported barriers highlighted the importance of literacy, trust, and sociocultural context in shaping digital health engagement.

Personal smartphone ownership in the present study was higher than what has been reported in national surveys of rural women in India, where fewer than half of women report personal phone use [17, 21]. This relatively higher access may be attributed to increasing affordability of smartphones, gradual normalization of mobile technology within rural households, and the presence of a functional primary healthcare system in the study area. However, this finding should not be interpreted as evidence of universal or equitable access, as a substantial proportion of women continued to rely on borrowed devices. Prior research has highlighted that shared phone use can limit privacy, autonomy, and consistency of access, particularly for women, thereby constraining the potential benefits of digital health interventions despite apparent device availability [14, 17].

Importantly, greater access did not translate into routine health-related use. While mobile phones were widely present, their use for managing health needs remained limited, underscoring the distinction between technological reach and meaningful engagement. Similar gaps between ownership and health-related use have been documented in other Indian and LMIC settings, where sociocultural norms, low digital literacy, and lack of confidence in navigating digital platforms reduce women’s independent use of mobile technology for health purposes [8, 14, 16]. These findings challenge the assumption that increasing phone penetration alone is sufficient to improve health communication and emphasize the need for interventions that address behavioral and contextual barriers.

Education emerged as the most consistent determinant of health-related mobile use and higher digital engagement. Women with lower levels of education were less likely to use phones for health purposes, even when access was available. This aligns with existing evidence demonstrating that education enhances digital literacy, comprehension of health information, and self-efficacy in interacting with technology [9, 17, 18]. In contrast, women with limited education may depend on intermediaries to interpret digital content, which can reduce both frequency of use and perceived relevance. These findings suggest that mHealth strategies relying heavily on text-based or app-driven content may inadvertently exclude the very populations they aim to reach unless supplemented with audio-based or assisted approaches.

Trust in mobile-delivered health information was generally conditional rather than absolute, reflecting continued reliance on interpersonal sources of credibility. Previous studies from India have consistently shown that frontline health workers, particularly ASHAs and ANMs, remain the most trusted sources of health information in rural communities [10, 12]. Digital messages that are perceived as disconnected from these trusted actors may be viewed with skepticism. Evidence from large-scale programs such as Kilkari suggests that mHealth interventions are more acceptable and effective when they complement, rather than replace, existing community-based communication channels [13]. The present findings reinforce the importance of integrating digital tools within established health worker networks to enhance trust and uptake.

Preferences for information delivery observed in the present study provide important insights for designing context-appropriate mobile health interventions. Voice calls emerged as the most preferred mode of receiving health-related information among rural women, suggesting that interactive and audio-based communication formats may be more acceptable than text-based messaging in populations with varying literacy levels. Similar observations from other rural and low-resource settings indicate that voice-based communication improves message comprehension and engagement compared to text-only approaches. These findings highlight the importance of tailoring digital health communication strategies to the literacy levels and communication preferences of target populations.

Perceived impact findings in the present study provide useful insights into the potential role of mobile phones in improving access to health-related information. Among women who reported using mobile phones for health purposes, a considerable proportion indicated improvement in access to healthcare information [13–15]. Although these perceptions do not establish causality, they suggest that mobile communication may function as a supportive tool that enhances awareness and facilitates timely healthcare seeking. Similar observations have been reported in studies evaluating community-based mobile health interventions in comparable rural settings [5, 11].

Overall, the findings underscore that while mobile phones offer significant opportunities to enhance health awareness among rural women, their effectiveness depends on addressing educational, social, and trust-related barriers. Future mHealth initiatives should prioritize inclusive design, integrate frontline health workers, and move beyond access metrics to evaluate meaningful engagement and impact. The relatively higher levels of smartphone access observed in this setting may reflect broader trends in increasing availability and affordability of mobile technologies in rural areas rather than specific characteristics of the local healthcare system.

### Policy implications

Taken together, these findings suggest that mobile phone ownership alone may not fully reflect digital health readiness, as meaningful engagement depends on literacy, trust, and consistent access to communication channels. The findings underscore the importance of strengthening digital literacy initiatives, promoting voice-based communication strategies, and integrating trusted community health workers into mobile health programs to improve adoption and effectiveness among rural women.

## Conclusion

This study demonstrates that although mobile phone access among rural women is relatively widespread, its translation into consistent and meaningful health-related use remains limited. Educational attainment strongly shapes digital engagement, while trust and preferred modes of communication favor interpersonal and voice-based approaches over text-based formats. These findings underscore the need for mHealth strategies that move beyond access metrics and address literacy, sociocultural context, and delivery preferences to achieve equitable health benefits.

## Recommendations

Future mHealth programs should prioritize voice-based and interactive communication, integrate frontline health workers to enhance trust, and incorporate targeted digital literacy initiatives for less educated women. Expanding content beyond maternal and child health to include chronic disease management may further improve relevance and uptake.

## Limitations and strengths

The study design limits causal inference, and self-reported responses may be subject to recall or social desirability bias. As the study was conducted in an RHTC field practice area, findings may not be fully generalizable to rural settings without similar health system exposure. This study provides a comprehensive assessment of mobile phone reach, use, and engagement among rural women using primary data. The inclusion of preferences, barriers, and a composite digital engagement score offers nuanced insights beyond ownership alone.

Additionally, the digital engagement score used in this study was developed by the authors and has not undergone formal external validation, which may limit comparability with other standardized digital engagement measures.

## Supplementary Information

Below is the link to the electronic supplementary material.


Supplementary Material 1


## Data Availability

The study datasets are coded and available from the corresponding author on reasonable request.

## References

[1] World Health Organization. mHealth: New horizons for health through mobile technologies. Geneva: WHO; 2011.

[2] Mechael PN. The case for mHealth in developing countries. Innov Technol Gov Glob. 2009;4(1):103–18.

[3] Labrique AB, Vasudevan L, Kochi E, Fabricant R, Mehl G. mHealth innovations as health system strengthening tools. Glob Health Sci Pract. 2013;1(2):160–71. 25276529 10.9745/GHSP-D-13-00031 PMC4168567

[4] Free C, Phillips G, Watson L, et al. The effectiveness of mobile-health technologies to improve health care service delivery processes. PLoS Med. 2013;10(1):e1001362. 23458994 10.1371/journal.pmed.1001363 PMC3566926

[5] Gurman TA, Rubin SE, Roess AA. Effectiveness of mHealth behavior change communication interventions in developing countries. J Health Commun. 2012;17(Suppl 1):82–104. 22548603 10.1080/10810730.2011.649160

[6] Lee SH, Nurmatov UB, Nwaru BI, Mukherjee M, Grant L, Pagliari C. Effectiveness of mHealth interventions for maternal, newborn and child health in LMICs. J Glob Health. 2016;6(1):010401. 26649177 10.7189/jogh.06.010401 PMC4643860

[7] Telecom Regulatory Authority of India. Indian Telecom Services Performance Indicators. New Delhi: TRAI; 2022.

[8] Aranda-Jan CB, Mohutsiwa-Dibe N, Loukanova S. Systematic review on barriers and facilitators to mHealth implementation. BMC Public Health. 2014;14:188. 24555733 10.1186/1471-2458-14-188 PMC3942265

[9] Crawford J, Larsen-Cooper E, Jezman Z, Cunningham SC, Bancroft E. SMS versus voice messaging to deliver MNCH information in rural Malawi. Glob Health Sci Pract. 2014;2(1):35–46. 25276561 10.9745/GHSP-D-13-00155 PMC4168599

[10] Scott K, George AS, Ved RR. Taking stock of ASHA programme implementation. Health Res Policy Syst. 2019;17(1):29. 30909926 10.1186/s12961-019-0427-0 PMC6434894

[11] Hall CS, Fottrell E, Wilkinson S, Byass P. Assessing the impact of mHealth interventions. Glob Health Action. 2014;7:25495. 10.3402/gha.v7.25606 PMC4216389 25361730

[12] George AS, Mehra V, Scott K, Sriram V. Community health workers at the interface of digital health. Health Policy Plan. 2021;36(2):258–71.

[13] Ved R, Gupta G, Ummer O, et al. Evaluation of Kilkari, India’s largest maternal messaging program. BMJ Glob Health. 2019;4(3):e001146.

[14] Chib A, van Velthoven MH, Car J. mHealth adoption in low-resource environments. J Health Commun. 2015;20(1):4–34. 24673171 10.1080/10810730.2013.864735

[15] Sinha RK, Nath A. Mobile phones as a tool for empowerment of rural women. Int J Health Sci Res. 2019;9(4):150–5.

[16] DeSouza SI, Rashmi MR, Vasanthi AP, Joseph SM, Rodrigues R. Mobile phones and healthcare delivery in rural India. J Fam Med Prim Care. 2019;8(9):2861–6. 10.1371/journal.pone.0104895 PMC4136858 25133610

[17] Mohan D, Bashingwa JJH, Tiffin N, Dhar D, Mulder N, George A. Does having a mobile phone matter? Glob Health Action. 2020;13(1):1830354.

[18] Rajan D, Thiruvengadam K, Kumar S. Digital health interventions for maternal health in India. Indian J Community Med. 2022;47(2):187–92.36034236

[19] Ministry of Health and Family Welfare. Government of India. Mobile Academy Programme. New Delhi: MoHFW; 2016.

[20] Ministry of Health and Family Welfare, Government of India. National Digital Health Mission: Strategy Overview. New Delhi: MoHFW; 2020.

[21] International Institute for Population Sciences (IIPS), MoHFW. National Family Health Survey (NFHS-5), 2019-21: India Fact Sheet. Mumbai: IIPS; 2021.

[22] International Institute for Population Sciences (IIPS), MoHFW. NFHS-5, 2019-21: Uttar Pradesh State Fact Sheet. Mumbai: IIPS; 2021.

[23] Lwanga SK, Lemeshow S. Sample size determination in health studies: A practical manual. Geneva: World Health Organization; 1991.

[24] Pandey VK, Aggarwal P, Kakkar R. Updated BG Prasad socioeconomic classification for the year 2025. Indian J Community Health. 2025;37(1):1–4.

