# Supplementary material for: Dialing into health: assessing the reach, use, and impact of mobile phones on health awareness among rural women in the Rural Health Training Centre area of District Gautam Buddha Nagar, Uttar Pradesh, India
Source: BMC Health Serv Res. 2026 Jun 20;26:1215. doi: 10.1186/s12913-026-15008-w (PMC13531852; doi:10.1186/s12913-026-15008-w)
Supplement: Supplementary file 1 [file 12913_2026_15008_MOESM1_ESM.docx]

**Supplementary Figure S1** Preferred modes of receiving health information among rural women

**Supplementary Table S1** Barriers to using mobile phones for health communication

| Barrier | n (%) |
| --- | --- |
| Lack of knowledge | 103 (27.5) |
| Language difficulty | 25 (6.8) |
| Family restrictions | 16 (4.4) |
| No network coverage | 5 (1.4) |
| Cost of data | 3 (0.8) |
| Other reasons | 221 (59.1) |
